# Physician burnout in Nigeria: a multicentre, cross-sectional study

**DOI:** 10.1186/s12913-020-05710-8

**Published:** 2020-09-14

**Authors:** Arinze D. G. Nwosu, Edmund N. Ossai, Uwakwe C. Mba, Ifeanyi Anikwe, Richard Ewah, Bernard O. Obande, Justin U. Achor

**Affiliations:** 1 Department of Anaesthesia, National Orthopaedic Hospital, Enugu, Nigeria; 2 Department of Community Medicine, College of Health Sciences, Ebonyi State University, Abakaliki, Nigeria; 3 Department of Plastic Surgery, College Of Medicine, ESUTH, Enugu, Nigeria; 4 Department of Orthopaedic Surgery, National Orthopaedic Hospital, Enugu, Nigeria; 5 Department of Anaesthesia, FETHA, Abakaliki, Nigeria; 6 Department of Orthopaedic Surgery, Federal Medical Centre, Makurdi, Nigeria; 7 Department of Psychiatry, Federal Neuropsychiatric Hospital, Enugu, Nigeria

**Keywords:** Burnout, Occupation, Physician, Patient safety, Oldenburg burnout inventory

## Abstract

**Background:**

Healthcare workers are a burnout-prone occupational group and the prevalence is particularly high among physicians. With the prevailing low physician-patient ratio in Nigeria which has worsened with the recent wave of physician emigration, among other socio-economic constraints; a setting for high physician burnout may have been nurtured. Our survey set out to determine the prevalence of burnout among physicians practicing in Nigeria, ascertain the factors that were associated with the development of burnout and evaluate the respondents’ perceived impact of physician burnout on patient safety.

**Methods:**

We used the Oldenburg burnout inventory as the measurement tool for burnout in the cross-sectional study conducted between November and December, 2019 among physicians in five tertiary health institutions in Nigeria. A 5- point Likert-type scale was used to evaluate the participants rating of their perceived impact of physician burnout on patient safety. Data entry and analysis were done using IBM Statistical package for social sciences software version 25 and the level of statistical significance was determined by a *p* value < 0.05.

**Results:**

The response rate was 61% (535/871), and burnout prevalence was 75.5% (404/535). Majority of the physicians (74.6%) perceive that physician burnout could impact patient safety. Physicians’ professional grade, age and years in practice, but not specialty, gender or marital status were associated with the exhaustion domain, whereas only the physicians’ age was associated with the disengagement domain of burnout. No socio-demographic or work-related characteristics determined overall burnout in our respondents.

**Conclusion:**

Physician burnout in Nigeria is high and pervasive, and this should alert physicians to be wary of their general and mental health status. Public health policy should address this development which has implications for patient safety, physician safety and healthcare system performance.

## Background

Burnout is a psychological syndrome that may develop when employees are exposed to a stressful working environment, with high job demands and low resources occurring simultaneously, resulting in exhaustion and disengagement; two related but conceptually distinct domains that constitute the core elements of burnout [1]. The service relationships that providers in the human services develop with recipients involve long hours of selfless service with intense level of personal and emotional contact. This pattern of interaction results in significantly higher levels of exhaustion and disengagement among health care workers compared to white collar workers as observed in a burnout evaluation among Dutch workforce [2]. Physicians are among the healthcare workers, but further constrained by statutory regulations and ethical considerations, excessive workload and huge clerical burden. Burnout is more common among physicians than other workers in the healthcare sector [3] and general population [4]. There is strong evidence that burnout is associated with poorer self-rated health and depression among healthcare workers [5]. Therefore physician burnout has crucial implication for their performance as well as the safety of their patient and self.

### Physician burnout and patient safety

There has been consistency in the association between burnout syndrome in healthcare workers and suboptimal and unsafe care of patients [6]. Major medical errors in surgical practice have been reported to be strongly related to the surgeon’s degree of burnout [7]. Self-reported suboptimal care of patients by physicians has also been attributed to burnout [8, 9]. Further evidence from a systematic review also supports that physician burnout is associated with an increased risk of patient safety incidents, poorer quality of care due to low professionalism, and reduced patient satisfaction [10].

### The Maslach burnout inventory (MBI) and Oldenburg burnout inventory (OLBI)

By far the MBI has been the predominant instrument for measuring burnout [11, 12] and has historically been regarded as the gold standard. According to Maslach, burnout consists of three dimensions: emotional exhaustion, depersonalization (felt distance from others), and diminished personal accomplishment. From this original version the MBI-Human services survey (MBI-HSS) [13] has evolved the MBI-General survey (MBI-GS) [14] based on the domains of exhaustion, cynicism and professional efficacy. However, whereas there is wide consensus on the multidimensional construct of burnout as against a uni-dimensional model, correlation between the personal accomplishment /professional efficacy dimension and the two others (Exhaustion and disengagement/ depersonalization/ cynicism) is low and this controversial component of the MBI has been questioned and challenged by several researchers [2, 15–18].

The OLBI is a validated instrument for measuring burnout [2, 19, 20] and there is growing evidence in support of its use [1, 19]. Convergence validity between the OLBI and MBI-GS has since been established [21]. The OLBI measures burnout with two dimensions: exhaustion and disengagement. In contrast to the MBI which includes only the emotional component of exhaustion, the OLBI makes provision for the physical and cognitive components of exhaustion. Furthermore, in the eight items of each domain of OLBI four items are positively worded while the four others are negatively worded, making for psychometric balancing.

Compounding the low physician-patient ratio and meager infrastructure in Nigeria, the country has witnessed massive emigration of her physician workforce overseas in recent years with the resultant creation of a healthcare system that could arguably drive physician burnout. Consequently, despite sparse literature on this occupational syndrome in Nigeria physician burnout may be pervading the practice space, albeit silently. We sought to determine the level of burnout among physicians in Nigeria, and the individual characteristics that are predictive of experiencing burnout.

## Methods

### Study design

A cross- sectional survey was conducted on physicians in five tertiary health institutions in Nigeria between November and December, 2019 using a pre-tested semi-structured questionnaire.

### Ethical approval

The Institutional review board of National Orthopedic Hospital, Enugu gave approval for the study and verbal informed consent to participate in the study was obtained from all the participating physicians.

### Sample population and participants

All physicians in all the departments of the enlisted health institutions were eligible for participation in the study. A total population sample of all the physicians in the five tertiary health institutions, located in three states of Nigeria; four in the southern part and one in the northern part of the country was carried out. Owing to their nature as training institutions and other inherent logistic weaknesses it was not possible to obtain the exact number of physicians in any of the centres as entry and exit into the various programs were constantly in a state of flux. In effect, the total number of physicians credited to each institution was the adjudged best estimate from the Association of Resident Doctors (ARD) and Medical and Dental Consultants Association of Nigeria (MDCAN) membership record; these being two broad groups to which all the physicians in the institutions belong. The study questionnaires were in print (paper) format and to minimize response bias they were anonymous and self-administered by the respondents.

### Survey administration

The questionnaire titled “A SOCIAL SURVEY OF FEELINGS AND ATTITUDES DURING WORK” comprised of three sections; (i) biodata section of the respondents (ii) a Likert-type scale of respondents rating of the impact of physician burnout on patient safety and (iii) the English version of the 16-item OLBI which is not so labeled. This generic labeling was adopted in an effort to fend off response bias to the tool, which might result if “burnout” were to be expressly mentioned. Each questionnaire is introduced with a definition of burnout thus; Burnout is a psychological syndrome that may result from exposure to a stressful working environment; with high job demands and low resources occurring simultaneously, resulting in exhaustion (general feeling of over-tasking from work) and disengagement (distancing oneself from ones work and negative attitude towards ones work). This is to enable the respondents give informed opinion of how they individually relate burnout to patient safety in the accompanying Likert -type tool. The tool for rating of influence of physician burnout on patient safety is framed thus “Rate your perception of physician burnout as being relevant to patient safety” with the following options; strongly disagree, disagree, undecided, agree, strongly agree.

### Survey tool -the OLBI instrument

Oldenburg burnout inventory is a 16-item survey with positively and negatively framed items which measure burnout with two dimensions: exhaustion (physical, cognitive, and affective aspects) and disengagement (negative attitudes toward work objects, work content, or work in general) [2]. In the 16-item version of the instrument used for this study each of these two dimensions is represented by 8 items; with four in each dimension being positively worded and four negatively worded and arranged in a mixed pattern for psychometric balancing. Items 2, 4, 5, 8, 10, 12, 14 and 16 explore exhaustion, while 1, 3, 6, 7, 9, 11, 13 and 15 explore disengagement. Each item has four Likert-type response options which are scored; strongly agree-1, agree-2, disagree-3, strongly disagree-4. Reverse scoring is applied to the items marked with an ‘R’ in the instrument such that strongly agree is scored-4 and strongly disagree-1. In all instances higher scores indicate higher exhaustion and disengagement.

### Data collection

The respondents were introduced to the survey during weekly departmental clinical meetings and outpatient clinic sessions. Repeat rounds of visits were made over three weeks by the research assistants to enable the recovery of questionnaires from those who could not complete theirs on the day of the initial distribution and to extend the distribution to those who were not reached previously.

### Outcome assessment

The main outcome assessed was burnout as measured by exhaustion and disengagement domain scores of the OLBI tool. Additional outcome measure assessed in the study was the respondents rating of physician burnout as relevant to patient safety; using the 5-item Likert-type template provided in the first section of the questionnaire with options graded as strongly disagree, disagree, undecided, agree, and strongly agree. For the purpose of analysis, the responses in the latter were dichotomized such that the responses of ‘agree’ and ‘strongly agree’ were regarded as YES while the responses of ‘strongly disagree’, ‘disagree’ and ‘undecided’ were regarded as NO with respect to the respondents perception of physician burnout as relevant issue to patient safety.

### Data analysis

Data entry and analysis were done using the statistical package for social sciences (SPSS) software version 25 (IBM, Armonk, New York, USA). Continuous variables were summarized using mean and standard deviation while categorical variables were summarized using proportions. For inferential statistics the Student t test was used to compare the difference in mean of two samples, Chi square test was used to compare difference in proportions and ANOVA was used to compare the difference in mean of more than two samples. The level of statistical significance was determined by a *p* value of < 0.05.

To identify the burnout groups, mean scores ≥2.25 on the exhaustion domain were considered as having high exhaustion, while those who scored less than 2.25 were regarded as having low Exhaustion. For the disengagement domain mean scores ≥2.1 were considered as high while those who scored less than 2.1 were regarded as having low Disengagement. These cut-off scores were adapted from a previous study on burnout among Swedish healthcare workers conducted with the OLBI [5]. The mean score for each domain is obtained by dividing the total scores for the items in the domain by the number of items in the domain; which is eight (8) in each case. The following categories emerged from the respondents;

- Burnout group: high exhaustion and high disengagement
- Exhausted group: high exhaustion and low disengagement
- Disengaged group: high disengagement and low exhaustion
- Non-burnout group: low disengagement and low exhaustion

In order to contend with the unwieldy number of specialties and subspecialties while considering the specialty of the physicians we applied the West African Postgraduate Medical College board classification such that; surgical specialties referred to general surgery, anesthesia, neurosurgery, cardiothoracic surgery, ophthalmology, otorhinolaryngology, pediatric surgery, maxillofacial surgery, orthopedic surgery, plastic surgery, pathology, radiology, obstetrics and gynecology while medical specialties referred to internal medicine, community medicine, psychiatry, family medicine and pediatrics. Medical officers and interns are non-specialists and as such were grouped as ‘Others’.

## Results

### Response rate

In National Orthopedic Hospital (NOHE), Enugu, out of 73 physicians 65 questionnaires were duly completed; in Federal Teaching Hospital, Abakaliki (FETHA) out of 430 physicians 186 questionnaires were completed; in Enugu State University Teaching Hospital (ESUTH) with 220 physicians 167 questionnaires were completed; in Federal Medical Centre (FMC), Makurdi with 124 physicians 97 questionnaires were completed, while in Federal Neuropsychiatric Hospital (FNPH), Enugu out of 24 physicians 20 completed questionnaires were returned: implying a response rate of 89, 43, 76, 78 and 83% respectively. In sum 535 duly completed questionnaires were returned out of a total physician population of 871, yielding a response rate of 61%.

### Participants perception of burnout impact on patient safety

In responding to their perception of physician burnout as relevant to patient safety; 74.6% (399/535) perceive physician burnout as relevant to patient safety, while 25.4% (136/535) disagreed.

Table 1 shows that males made up 73.8% of the respondents, married physicians 72.1%, and resident doctors 59.4%. The male and female physicians had the same mean exhaustion score, 2.7 ± 0.4 and mean disengagement score of 2.4 ± 0.4.

**Table 1:** Socio-demographic characteristics of respondents

Variable	Frequency (***n*** = 535)	Exhaustion Mean(±SD)	Disengagement Mean ± SD
**Age of respondents in groups**			
< 35 years	195 (36.4)	2.7 ± 0.4	2.4 ± 0.4
35–39 years	158 (29.5)	2.7 ± 0.4	2.5 ± 0.4
≥ 40 years	182 (34.0)	2.6 ± 0.4	2.4 ± 0.4
**Gender**			
Male	395 (73.8)	2.7 ± 0.4	2.4 ± 0.4
Female	140 (26.2)	2.7 ± 0.4	2.4 ± 0.4
**Marital status**			
Married	386 (72.1)	2.7 ± 0.4	2.4 ± 0.4
Single	149 (27.9)	2.7 ± 0.4	2.4 ± 0.4
**Number of years in practice**			
< 5 years	148 (27.7)	2.8 ± 0.4	2.4 ± 0.4
5–9 years	134 (25.0)	2.7 ± 0.4	2.4 ± 0.4
10–14 years	164 (30.7)	2.6 ± 0.5	2.4 ± 0.4
≥ 15 years	89 (16.6)	2.5 ± 0.4	2.3 ± 0.4
**Professional grade of physicians**			
Medical consultant	79 (14.8)	2.5 ± 0.4	2.3 ± 0.4
Medical officer	22 (4.1)	2.7 ± 0.5	2.4 ± 0.5
Resident doctor	318 (59.4)	2.7 ± 0.4	2.4 ± 0.4
Medical intern	116 (21.7)	2.7 ± 0.4	2.4 ± 0.4
**Institution**			
NOHE, Enugu.	65 (12.1)	2.6 ± 0.4	2.4 ± 0.4
FETHA, Abakaliki.	186 (34.8)	2.6 ± 0.4	2.4 ± 0.4
ESUTH, Enugu.	167 (31.2)	2.7 ± 0.4	2.4 ± 0.4
FMC, Makurdi.	97 (18.1)	2.7 ± 0.4	2.4 ± 0.4
FNPH, Enugu.	20 (3.7)	2.6 ± 0.5	2.3 ± 0.4
**Medical specialty of respondents**			
Surgical specialties	261 (48.8)	2.7 ± 0.4	2.4 ± 0.4
Medical Specialties	136 (25.4)	2.6 ± 0.4	2.4 ± 0.4
Others*	138 (25.8)	2.7 ± 0.4	1 2.4 ± 0.4

*No specific specialty

Table 2, shows that the difference in the mean of exhaustion domain scores is statistically significant with respect to the physicians’ professional grade, age, and years in practice.

**Table 2:** Association between exhaustion scores and respondents characteristics

Variable	(***n*** = 535)	Exhaustion Mean (±SD)	F	***p*** value
**Professional grade of physicians**				
Medical consultant	79	2.5 ± 0.4	6.793	< 0.001
Medical officer	22	2.7 ± 0.5		
Resident doctor	318	2.7 ± 0.4		
Medical intern	116	2.7 ± 0.4		
**Age group of physicians**				
< 35 years	195	2.7 ± 0.4	10.734	< 0.001
35–39 years	158	2.7 ± 0.4		
≥ 40 years	182	2.6 ± 0.4		
**Years in practice**				
< 5 years	148	2.8 ± 0.4	9.317	< 0.001
5–9 years	134	2.7 ± 0.4		
10–14 years	164	2.6 ± 0.5		
≥ 15 years	89	2.5 ± 0.4		
**Specialties**				
Surgical specialties	261	2.7 ± 0.4	1.811	0.164
Medical specialties	136	2.6 ± 0.4		
Others*	138	2.7 ± 0.4		
**Gender**				
Male	395	2.7 ± 0.4	1.616**	0.107
Female	140	2.7 ± 0.4		
**Marital status**				
Married	386	2.7 ± 0.4	1.792**	0.074
Single	149	2.7 ± 0.4		

*No specific specialty **Student t test

From Table 3, we observed that the medical officers had the highest mean disengagement score, 2.4 ± 0.5 while the medical consultants had the least, 2.3 ± 0.4 but the difference in mean was not statistically significant, (F = 2.091, *p* = 1.0). The respondents in the age group, 35–39 years had the highest mean disengagement score, 2.5 ± 0.4 and the difference in mean was statistically significant, (F = 3.183, *p* = 0.042).

**Table 3:** Association between disengagement scores and respondents characteristics

Variable	(***n*** = 535)	Disengagement Mean (±SD)	F	***p*** value
**Professional grade of physicians**				
Medical consultant	79	2.3 ± 0.4	2.091	1.0
Medical officer	22	2.4 ± 0.5		
Resident doctor	318	2.4 ± 0.4		
Medical intern	116	2.4 ± 0.4		
**Age groups of physicians**				
< 35 years	195	2.4 ± 0.4	3.183	0.042
35–39 years	158	2.5 ± 0.4		
≥ 40 years	182	2.4 ± 0.4		
**Years in practice**				
< 5 years	148	2.4 ± 0.4	1.638	0.180
5–9 years	134	2.4 ± 0.4		
10–14 years	164	2.4 ± 0.4		
≥ 15 years	89	2.3 ± 0.4		
**Specialties**				
Surgical specialties	261	2.4 ± 0.4	0.785	0.457
Medical specialties	136	2.4 ± 0.4		
Others*	138	2.4 ± 0.4		
**Gender**				
Male	395	2.4 ± 0.4	0.025**	0.980
Female	140	2.4 ± 0.4		
**Marital status**				
Married	386	2.4 ± 0.4	0.933**	0.351
Single	149	2.4 ± 0.4		

*No specific specialty **Student t test

The highest proportion of respondents, 75.5% were in the burnout group (Table 4.).

**Table 4:** Burnout category of the physicians

Variable	Frequency (***n*** = 535)	Percent (%)
**Burnout grouping**		
Non Burnout group	34	6.4
Disengaged group	27	5.0
Exhausted group	70	13.1
Burnout group	404	75.5

There was no association between burnout and hospital characteristics of the respondents (χ^2^ = 1.373, *p* = 0.849) and comparable proportions of male (76.7%) and females respondents (75.0%) were in the physician burnout group, (χ^2^ = 0.027, *p* = 0.869).

## Discussion

We found that 75.5% of the physicians surveyed were burnt-out, but no individual or institutional predictors of overall burnout were identified. A majority of the physicians (74.6%) perceive that physician burnout could impact patient safety.

While there is paucity of studies examining physicians’ perception of burnout’s impact on patient safety, a majority of burnout-prone healthcare workers surveyed in a tertiary health institution in Rwanda aver to suboptimal patient care, medical errors and poor outcome which they attributed to burnout [22].

The overall response rate recorded in the study (61%) was comparable to that obtained in two previous studies on physician burnout in Nigeria [23, 24]. Nevertheless the response rate of 43% in one of the institutions (FETHA, Abakaliki) was disproportionately low compared to the other institutions. The institution is a relatively new one with a significant number of physicians being primarily employed elsewhere. High response rate and the mode of questionnaire administration (paper, self-administered) used in this study have been found to minimize bias and positively impact on the quality of data generated and the validity of the results of a research [25].

The high prevalence of burnout found in this study (75.5%) is supported by the report of a meta-analysis of physician burnout across global regions (which lamented paucity of research on physician burnout in Africa) that found physician burnout to be positively associated with workload, constraining organizational structure, violence and low safety standards [26]; all of which characterize the current work environment of the physician practicing in Nigeria. There had been few published works on physician burnout in Nigeria. One of them, a small survey of orthopedic surgeons had reported a burnout prevalence of 51.7% (15/29) [27]. Two other Nigerian studies are equally very limited in scope; being restricted to primary care physicians in a city [23], and resident doctors in a single institution [24]. All three studies were carried out between 2010 and 2015; a period predating the current wave of Nigerian physician emigration to Europe and the Middle East. Among the few studies on physician burnout conducted elsewhere in Africa, prevalence of 81 and 76% respectively were reported among physicians in South African rural district hospitals and urban hospitals respectively [28, 29]. In a 2017 study of all physicians working in all the public hospitals (primary, secondary and tertiary) in Southern Ethiopia, burnout was pervasive; with 65.2% of physicians scoring high on emotional exhaustion, 85.1% showed high degree of depersonalization and 91% of them experienced low level of personal accomplishment [30]. A systematic review of burnout among physicians in the UK conducted in 2013 rated high emotional exhaustion scores in the range of 31 to 54.3%; high depersonalization scores ranged from 17.4 to 44.5% and for low personal accomplishment the range was 6–39.6% [31].

Our study revealed a strong association between the exhaustion domain of burnout and professional grade, age and years in practice of the physician (see Table 2), while the only association found in respect of the disengagement domain was with the age group of the physicians (see Table 3). These associations were however only applicable as far as the independent domains of burnout are considered, but none of these or other socio-demographic and work-related characteristics were found to predict overall burnout in our respondents (see Table 5). This inability to locate any socio-demographic predictor of burnout in the respondents may suggest that factors external to the individual physicians – system factors, are responsible. This postulation is supported by the findings of a meta-analysis that evaluated the effectiveness of different types of interventions to reduce burnout in physicians [32]. Thus with the greater improvement credited to organization-directed interventions compared with physician-directed interventions in the meta-analysis it concluded that organizational factors, more than individual factors, drive physician burnout. The finding of similar burnout indices in the different health institutions surveyed may thus reflect the pervasive and universal nature of the burnout-promoting practice environment in the country. A UK study to assess the prevalence of psychological morbidity across different surgical specialties revealed that burnout prevalence did not differ significantly between specialty, professional grade, age, or gender of the surgeon, but that the number of years in the specialty independently predicted scores in the exhaustion domain [33]. Our study did not find any association between physician’s specialty and burnout. This may have derived from the fact that the groups as we composed are a cocktail of specialties with disparate and paradoxical attributes in training and practice experience. Furthermore physician interns are a large group with ubiquitous representation in the different specialties in the course of their rotations, as such would not be justifiably allocated to either surgical or medical specialty owing to their mixed exposure and experience. A survey of residents and interns in dermatology, general surgery, internal medicine, family medicine, neurology, obstetrics/gynecology, ophthalmology, and psychiatry departments in a US tertiary health institution reported burnout rates ranging from 75% in obstetrics/gynecology to 27% in family medicine [34]. Another US- based study by Shanafelt et al., [4] reported the highest burnout rates among emergency medicine physicians with lowest rates in preventive medicine and dermatology, but this specialty trend showed significant variation in a subsequent national survey [12]. Meanwhile elsewhere a multicentre survey in Italy found that psychiatrists had higher levels of burnout than other physicians [35].

**Table 5:** Determinants of burnout among the physician

Variable	Physician Burnout (***n*** = 535)		χ^**2**^	*p* value
	Yes ***N*** (%)	No ***N*** (%)		
**Age of respondents in groups**				
< 38 years	227 (77.2)	67 (22.8)	1.016	0.313
≥ 38 years	177 (73.4)	64 (26.6)		
**Gender**				
Male	299 (76.7)	96 (24.3)	0.027	0.869
Female	105 (75.0)	35 (25.0)		
**Marital status**				
Married	288 (74.6)	98 (25.4)	0.611	0.435
Single	116 (77.9)	33 (22.1)		
**Number of years in practice**				
< 10 years	221 (78.4)	61 (21.6)	2.628	0.105
≥ 10 years	183 (72.3)	70 (27.7)		
**Professional grade of physicians**				
Medical consultant	54 (68.4)	25 (31.6)	3.453	0.327
Medical officer	16 (72.7)	6 (27.3)		
Resident doctor	248 (78.0)	30 (25.9)		
Medical intern	86 (74.1)	30 (25.9)		
**Institution**				
NOHE, Enugu.	47 (72.3)	18 (27.7)	1.373	0.849
FETHA, Abakaliki.	137 (73.7)	49 (26.3)		
ESUTH, Enugu.	130 (77.8)	37 (22.7)		
FMC, Makurdi	75 (77.3)	22 (22.7)		
FNPH, Enugu.	15 (75.0)	5 (25.0)		
**Perception of burnout as relevant to patient safety**				
Yes	305 (76.4)	94 (23.6)	0.730	0.393
No	99 (72.8)	37 (27.2)		
**Specialties**				
Surgical specialties	201 (77.0)	60 (23.0)	0.623	0.732
Medical specialties	101 (74.3)	35 (25.7)		
Others*	102 (73.9)	36 (26.1)		

One major challenge with the prevalence of burnout quoted by many researchers reporting their work with other burnout instruments such as the MBI has been the highly variable criteria that qualify burnout. It is commonplace to find low prevalence with studies in which the criteria is the simultaneous occurrence of high burnout in all the three subscales of exhaustion, depersonalization and low personal accomplishment; such as 4.2% [36], 8.5% [37] and 11.7% [38]. In a study of burnout amongst physicians working in primary care in Saudi Arabia, out of 144 respondents; 48 (33.3%) scored high burnout in only one subscale, 57 (39.6%) scored high burnout in two subscales while only 4(2.78%) scored high burnout in **all** three subscales. They defined high burnout as concurrent high scores in emotional exhaustion (≥27), high scores in depersonalization (≥10) and low scores in personal accomplishment (≤33) and based on this criterion burnout prevalence was reported as 2.78% [39]. Had they used the criterion that recognizes burnout as concurrent occurrence of high burnout scores in two subscales, the same study may have posted a burnout prevalence of 39.6% instead of 2.78%. This wide variability in the defining criteria applied by different researchers has elicited concern in a systematic review which revealed five different criteria applied by different researchers, and the attendant implication to burnout prevalence reports [40]. Consequently with the variation in burnout estimation criteria by different researchers it was difficult to make direct comparison between the results of our study and other studies.

The high prevalence of burnout in this study as measured by the OLBI is comparable to other reports from Africa where an alternative tool (MBI) was used. It is supported by the findings of earlier research on correlates of physician burnout across global regions [26] and the twin problem of physician exodus and the prevailing environment of healthcare in Nigeria. The current wave of physician exodus in Nigeria also supports our burnout prevalence as several researchers have reported a remarkable association between burnout among physicians and the intention to emigrate [41, 42].

Against the background of very high burnout among physicians in Nigeria there is justification for further studies to determine its impact on the physicians’ health and well-being. Longitudinal studies on Nigerian physician emigrants would be necessary to validate the role of system and environmental factors in the high burnout observed at home. Furthermore, implementation of structured system interventions and evaluation of their effect on mitigating burnout among Nigerian physicians are essential, and could represent a robust effort at controlling burnout.

## Limitations

As indicated earlier in the methodology section we were unable to obtain the accurate data on the number of eligible physicians in the respective training institutions. Furthermore, despite the multi-centre and multispecialty design of this study the institutions surveyed are government-owned tertiary health institutions which are located in urban areas. Though these facilities also provide primary and secondary care the findings may not necessarily reflect the scenario in private institutions or health facilities located in the rural areas. This consideration may not be ignored despite the finding of similar trends in a comparative study of physician burnout in South African rural and urban hospitals [28, 29]. Response bias is a known shortcoming of convenient sampling technique which we applied in this study and this could have had some influence on the findings.

## Conclusion

Physician burnout in Nigeria is high and pervasive, and this should alert physicians to be wary of their general and mental health status. Public health policy should address this development which has implications for patient safety, physician safety and healthcare system performance; as the goal of safe and efficient care would remain a mirage with a very unhealthy population of physicians.

## Data Availability

The datasets used and analyzed during the current study are available from the corresponding author on reasonable request.

## Abbreviations

ANOVA: Analysis of variance
ARD: Association of resident doctors
ESUTH: Enugu State University Teaching Hospital
FETHA: Federal Teaching Hospital, Abakaliki
FMC: Federal Medical Centre
FNPH: Federal Neuropsychiatric Hospital
GP: General practitioners
MBI: Maslach burnout inventory
(MBI-GS): MBI-General survey
(MBI-HSS): MBI-Human services survey
MDCAN: Medical and Dental consultants association of Nigeria
NHS: National Health Service
NOHE: National Orthopedic Hospital, Enugu
OLBI: Oldenburg burnout inventory
SPSS: Statistical package for social sciences
UK: United Kingdom
